# Sandwich ELISA for the Quantification of Nucleocapsid Protein of SARS-CoV-2 Based on Polyclonal Antibodies from Two Different Species

**DOI:** 10.3390/ijms25010333

**Published:** 2023-12-26

**Authors:** Maja Mladenovic Stokanic, Ana Simovic, Vesna Jovanovic, Mirjana Radomirovic, Bozidar Udovicki, Maja Krstic Ristivojevic, Teodora Djukic, Tamara Vasovic, Jelena Acimovic, Ljiljana Sabljic, Ivana Lukic, Ana Kovacevic, Danica Cujic, Marija Gnjatovic, Katarina Smiljanic, Marija Stojadinovic, Jelena Radosavljevic, Dragana Stanic-Vucinic, Marijana Stojanovic, Andreja Rajkovic, Tanja Cirkovic Velickovic

**Affiliations:** 1 Centre of Excellence for Molecular Food Sciences, Department of Biochemistry, Faculty of Chemistry, University of Belgrade, Studentski trg 12-16, 11000 Belgrade, Serbia; 2 Department of Food Safety and Quality Management, Faculty of Agriculture, University of Belgrade, Nemanjina 6, Zemun, 11080 Belgrade, Serbia; 3 Institute of Medical Chemistry, Faculty of Medicine, University of Belgrade, Višegradska 26, 11000 Belgrade, Serbia; 4 Institute for the Application of Nuclear Energy—INEP, University of Belgrade, Banatska 31b, Zemun, 11080 Belgrade, Serbia; 5 Institute of Virology, Vaccines, and Sera–TORLAK, Vojvode Stepe 458, 11152 Belgrade, Serbia; 6 Department of Molecular Biology, Institute for Biological Research “Siniša Stanković”, University of Belgrade, 142 Despot Stefan Blvd., 11000 Belgrade, Serbia; 7 Faculty of Bioscience Engineering, Ghent University, Coupure Links 653, geb. A, B-9000 Ghent, Belgium; 8 Serbian Academy of Sciences and Arts, Kneza Mihaila 35, 11000 Belgrade, Serbia; 9 Global Campus, Ghent University, 119-5 Songdomunwha-ro, Yeonsu-gu, Incheon 21985, Republic of Korea

**Keywords:** COVID-19 diagnosis, nucleocapsid protein, antigen test, ELISA, polyclonal antibodies

## Abstract

In this study, a cost-effective sandwich ELISA test, based on polyclonal antibodies, for routine quantification SARS-CoV-2 nucleocapsid (N) protein was developed. The recombinant N protein was produced and used for the production of mice and rabbit antisera. Polyclonal N protein-specific antibodies served as capture and detection antibodies. The prototype ELISA has LOD 0.93 ng/mL and LOQ 5.3 ng/mL, with a linear range of 1.52–48.83 ng/mL. N protein heat pretreatment (56 °C, 1 h) decreased, while pretreatment with 1% Triton X-100 increased analytical ELISA sensitivity. The diagnostic specificity of ELISA was 100% (95% CI, 91.19–100.00%) and sensitivity was 52.94% (95% CI, 35.13–70.22%) compared to rtRT-PCR (Ct < 40). Profoundly higher sensitivity was obtained using patient samples mostly containing Wuhan-similar variants (Wuhan, alpha, and delta), 62.50% (95% CI, 40.59 to 81.20%), in comparison to samples mostly containing Wuhan-distant variants (Omicron) 30.00% (6.67–65.25%). The developed product has relatively high diagnostic sensitivity in relation to its analytical sensitivity due to the usage of polyclonal antibodies from two species, providing a wide repertoire of antibodies against multiple N protein epitopes. Moreover, the fast, simple, and inexpensive production of polyclonal antibodies, as the most expensive assay components, would result in affordable antigen tests.

## 1. Introduction

Although the state of COVID-19 infection is endemic rather than pandemic, there is still a need for a quantitative assessment of the SARS-CoV-2 viral load, particularly for guiding therapy to high-risk patients. The presence of SARS-CoV-2 can be confirmed by real-time reverse transcription polymerase chain reaction (rtRT-PCR) for the detection of viral RNA or by using ELISA and similar antibodies-based techniques that detect viral antigens in biological fluids. rtRT-PCR is the gold standard diagnostic test for COVID-19 as it is highly specific and sensitive. However, it suffers from false-negative results, mainly due to viral RNA degradation and loss during the transport, storage, and handling of the samples. Moreover, the detection of viral RNA remnants long after the virus had disappeared in rtRT-PCR false-positive results. In addition, SARS-CoV-2 RNA in the upper respiratory tract is not a reliable marker of infection severity since high concentrations of the virus in nasopharyngeal and saliva specimens are observed also in asymptomatic and mild SARS-CoV-2 infections [1]. Moreover, rtRT-PCR requires the use of sophisticated equipment and well-trained staff and takes many steps to be completed [2].

In contrast, antigen detection using immunoassays can be easily performed and interpreted; in addition, they are inexpensive, have shorter turnaround times, and correlate better with patient infectiousness than rtRT-PCR results [3]. Antibody-based tests (immunoassays) for the detection of SARS-CoV-2 antigens could complement rtRT-PCR tests to enhance the overall sensitivity of testing and reduce false-negative and false-positive rates, providing reliable and timely SARS-CoV-2 diagnoses of acute infection and recovering. Moreover, antigen-based tests may serve as an alternative tool for the early diagnosis of SARS-CoV-2 infection in laboratories with limited resources and expertise, as well as mass screening for the reservoir of the virus. 

Different assays were designed to target the SARS-CoV-2 nucleocapsid (N) protein as an antigen, due to several reasons: the large copy number of N protein per viral particle, (~1000) reaching up to ~1% of expressed proteins during infection [4], is less prone to mutation in comparison to spike (S) protein, and it is also highly immunogenic. Moreover, the presence of N protein is a better marker for viral replication than rtRT-PCR since N protein is associated with cellular membranes, and it is nuclease resistant and thus can be detected even after active replication has stopped [5]. N protein can be detected during active SARS-CoV-2 infection after two days of symptoms, and, during the first week, the infection detection of N in plasma has a sensitivity of >90%, where N protein concentration positively correlates to disease severity [6,7]. 

ELISA-based antigen detection tests are well known to offer high specificity and reproducibility; they are easy to acquire and standardize and are free of the problem of contamination [8]. N protein-specific ELISAs can test a variety of patient sample types while achieving the levels of sensitivity and specificity required for effective community screening [9]. 

The majority of commercially available antigenic tests for SARS-CoV-2 are based on monoclonal anti-N antibodies. Monoclonal antibodies give less background, ensure reproducible results due to homogeneity, and are highly specific as they target only one epitope that could be selected in a way to minimize cross-reactivity. In contrast, polyclonal antibodies recognize multiple epitopes, provide more robust detection, and have higher tolerance for slight differences in antigen structure. Moreover, in sandwich ELISA usage, polyclonal antibodies raised in different animal species can further increase the chances of analyte detection. Moreover, the production of polyclonal antibodies is less demanding and several times cheaper than the production of monoclonal antibodies. It should be mentioned that for every batch of polyclonal antibodies, a calibration of the assay must be performed, slightly increasing costs to the assay; regardless, the assay is still far cheaper than an assay based on monoclonal antibodies.

Virus inactivation can be accomplished via physical (heat, ultraviolet light, sonication, and ionizing radiation) and chemical (detergents, fixatives, and denaturants) methods, or by their combinations [10]. The virus inactivation could have a significant impact on the sensitivity of antigen assays, especially for low viral load samples [11]. Recent studies have shown that heat (56 °C for 30 min), detergent (1–5% Triton X-100), and solvent−detergent combinations (0.3–1% tri-n-butyl phosphate and 1–2% Triton X-100) are deemed immunoassay compatible when the average and range of percentage recovery (treated concentration relative to untreated concentration) lie between 90−110% and 80–120%, respectively [12].

The objective of this study was to establish a sandwich ELISA for the quantification of SARS-CoV-2 N protein using polyclonal antibodies as capturing and detecting reagents, thus being cost-effective and recognizing multiple epitopes. ELISA was analytically and diagnostically validated compared to rtRT-PCR. In addition, the effects of heat treatment (56 °C) and the addition of detergent (1% Triton X-100) on analytical and diagnostic sensitivity were examined.

## 2. Results

### 2.1. Production of N Protein and Anti-N Protein-Polyclonal Antibodies

#### 2.1.1. Preparation of Recombinant N Protein Fragment and Post-Translational Modification (PTM) Profiling

The purity and PTM profile of expressed and purified rfNP, henceforth annotated as N protein, was checked (Section S1, Figure S1) and analyzed using SDS PAGE followed by tandem mass spectrometry. After the in-gel digestion of the excised band at 40 kDa, the tandem mass spectrometry identification of proteins confirmed the identity of N protein with high scores and peptide coverage above 70% (Section S1, Figure S2 and Table S1).

#### 2.1.2. Antisera Production and Polyclonal Antibody Characterization

**Figure 1:**
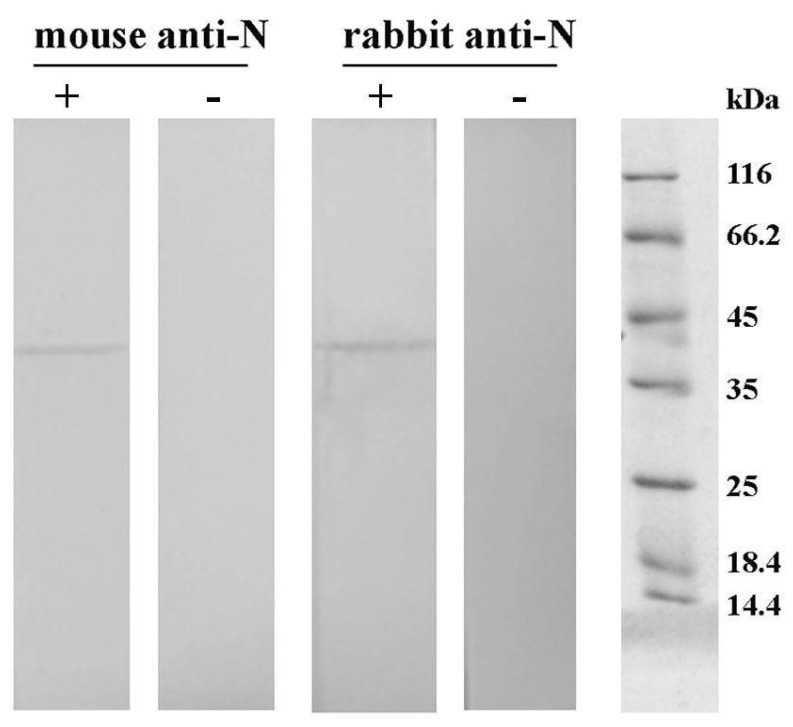
Western blot analysis of N protein probed with purified mouse and rabbit antiserum, followed by incubation with either biotinylated goat anti-mouse IgG antibodies and streptavidin conjugated with alkaline phosphatase (AP) or goat anti-rabbit IgG-AP conjugate (+). Secondary antibody controls (−) were also included.

For the development of an ELISA test specific for the detection of SARS-CoV-2 N protein, recombinantly produced N protein was used for the immunization of mice and rabbits. Collected mice and rabbit sera showed high titers of antibodies against N protein, and their purification using AS precipitation followed by affinity chromatography on protein A Sepharose resulted in highly purified antibodies (Section S2, Figure S3). A Western blot analysis of N protein probed with purified mice and rabbit antibodies showed only one protein band on mass around 40 kDa, confirming that the obtained purified polyclonal antibodies are strong binders of produced recombinant N protein (Figure 1).

### 2.2. Capture ELISA Prototype and Its Analytical Validation

**Figure 2:**
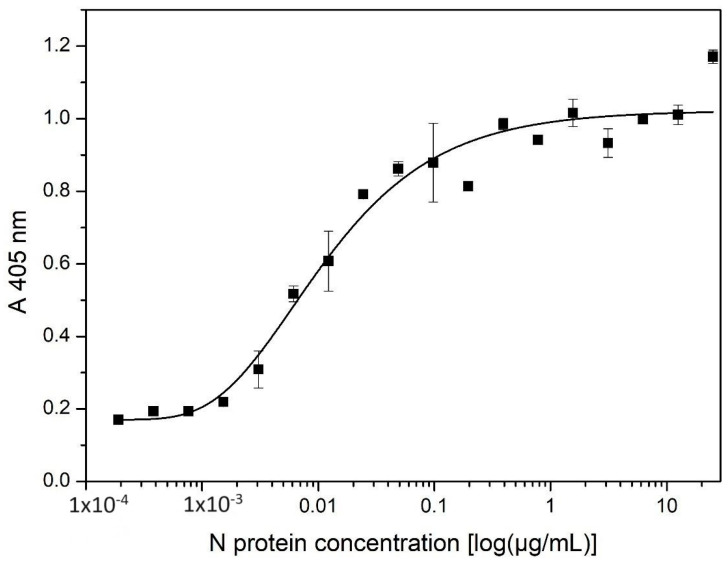
Representative calibration curve for quantification of N protein by developed prototype ELISA.

Capture ELISA was developed using rabbit polyclonal antibodies as capture antibodies, as well as mice antibodies as detection antibodies. The standard curve represents the dependence of A405 on N protein concentration (Figure 2).

#### 2.2.1. Analytical Sensitivity and Linear Range

The LOD value was determined to be 0.931 ng/mL, while the LOQ value was 2.124 ng/mL. The linear range of the developed ELISA was in the 1.52–48.83 ng/mL range, with a coefficient of determination of 0.992.

#### 2.2.2. Intra- and Inter-Day Precision

To assess the reproducibility of the ELISA, intra-day and inter-day variabilities were evaluated with ten fixed dilutions of N protein, 0.78, 1.56, 3.12, 6.25, 12.5, 25, 50, 100, 200, and 400 ng/mL (Table 1). The intra-day variability was determined for three ELISAs performed independently during the same day, and inter-day variability was determined for three ELISAs performed on three different days.

CV% of A405 values for intra-day ranged from 1.51 to 20.69%; meanwhile, inter-day CV% ranged from 11.33 to 46.03%. These results demonstrate that the precision of our developed ELISA is high in a broad concentration range covering more than three orders of magnitude, considering that, generally, CV% between 10 and 20% is as good and CV% < 10% is very good. A higher CV% was obtained for the lowest N protein concentrations for both inter- and intra-day precision testing.

#### 2.2.3. ELISA Accuracy

The accuracy of the developed ELISA was validated based on the recovery of N protein from three biological matrices spiked with N protein. SSF, 6% HSA, and AU were spiked with N protein at 100, 25, and 6.25 ng/mL of the biological matrix.

The highest recovery was obtained with the highest spiking dose (81.37–105.72%), while the lowest recovery was with the lowest spiking dose (39.70–61.08%) (Table 2). CV% ranged between 0.83 and 71.66%. For 6% HSA, at all spiked concentrations, CV% was <20%; meanwhile, AU CV% was the highest among the tested matrices. In simulated plasma, which is the simple solution of HSA in PBS, we expected to obtain the highest accuracy. SSF is a relatively simple solution of highly soluble inorganic salts, while AU is a more complex solution containing organic acids/salts (oxalate, citrate, and uric acid) and urea, in addition to inorganic salts, including Ca^2+^ ions. Thus, a high CV% was expected in AU as a matrix.

#### 2.2.4. Testing of Different Conditions for Sample Pretreatment before ELISA

To investigate the effects of different SARS-CoV-2 inactivation procedures on the sensitivity of the ELISA, N protein was quantified with a developed ELISA in five positive samples (confirmed using rtRT-PCR) subjected to thermal, chemical, and thermal-chemical pretreatment. Untreated samples were prepared through the dilution of leftovers from rtRT-PCR with blocking buffer at a ratio of 1:1 (*v*/*v*), followed by incubation at room temperature. Thermally treated samples were prepared in the same way, except that incubation was performed at 56 °C for 1 h. Chemically treated samples were prepared as untreated, except that the blocking buffer contained 2% Triton X-100. Chemically and thermally treated samples were diluted by the blocking buffer with 2% Triton X-100 (1:1, *v*/*v*), followed by 1 h incubation at 56 °C.

**Figure 3:**
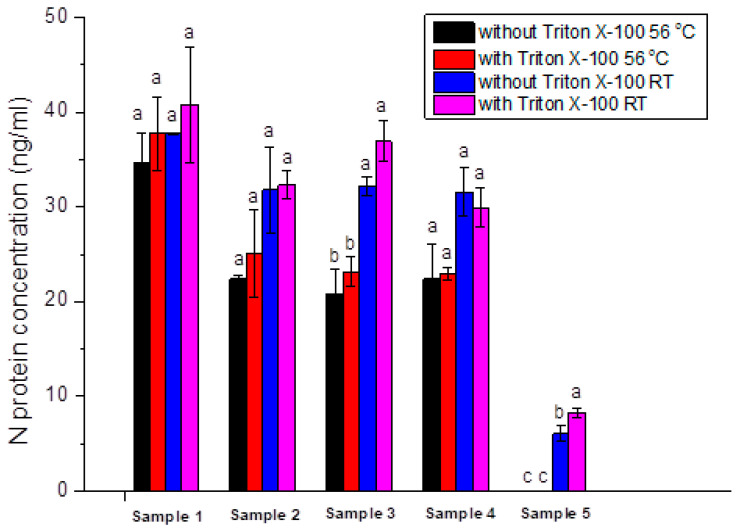
N protein quantification in five rtRT-PCR-positive samples after different chemical and thermal sample pretreatments: no treatment (without Triton X-100, 1 h at room temperature), chemical treatment (with Triton X-100, 1 h at room temperature); thermal treatment (without Triton X-100, 1 h at 56 °C) and combined chemical and thermal treatment (with Triton X-100, 1 h at 56 °C). Statistical significance (one-way ANOVA with Tukey’s multiple comparisons at *p* < 0.05) was determined for the effects of different treatments within one sample, and the same letter annotations mean no statistically significant difference while different letter annotations represent statistically significant results.

Figure 3 demonstrates that thermal treatment negatively influenced assay sensitivity. For sample 2, the thermal treatment tends to decrease assay sensitivity, and, for samples 3 and 5, this decrease is statistically significant (*p* < 0.05). Chemical treatment with Triton X-100 tends to increase assay sensitivity for samples 2 and 3, and, for sample 5, this increase is statistically significant (*p* < 0.05). Therefore, the diagnostic validation of the N protein ELISA was performed with and without sample pretreatment with Triton X-100.

### 2.3. Diagnostic Validation of N Protein ELISA

Diagnostic validation of the developed N protein ELISA was performed by determining diagnostic sensitivity, specificity, compatibility (accuracy), positive predictive value (PPV), and negative predictive value (PPV) calculated in comparison with rtRT-PCR as reference method. The rtRT-PCR reference method used has a sensitivity of 200 copies/mL (with Ct < 40) and has no cross-reactivity with other coronaviruses and respiratory pathogens (https://www.fda.gov/media/137651/download) (accessed on 1 September 2023).

Clinical validation was performed using 76 nasopharyngeal swabs, of which 34 (46%) samples were positive and 0 (54%) were negative, as determined using the rtRT-PCR reference method (Section S3, Figure S4). Samples for which Ct ≤ 40 were considered as positive. In parallel, all samples were analyzed using the developed N protein ELISA, where the samples were considered positive with an N protein concentration > LOD. 

The diagnostic validation was performed using plates coated with capture antibody stabilized with 3% sucrose and 10% glycerol and stored for 3 weeks (Section S3.1). In parallel with the samples’ LOD, a plate with a stabilized capture antibody that was stored for 3 weeks was determined, and this LOD (1.00 ng/mL) was used to determine ELISA-positive samples.

**Figure 4:**
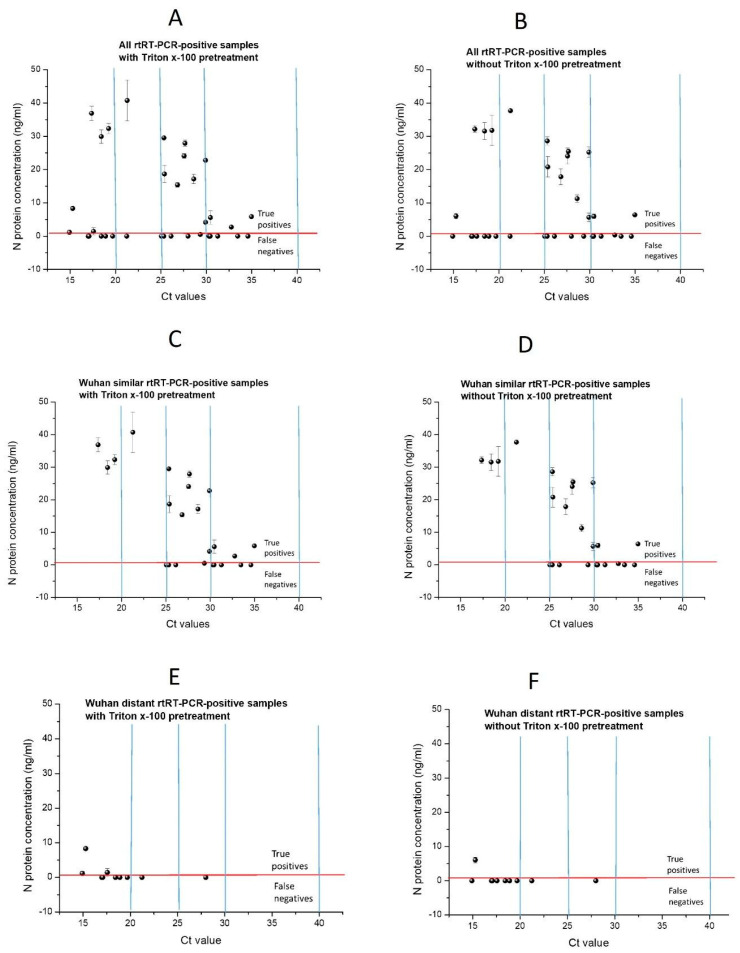
Distribution of N protein concentration in rtRT-PCR-positive samples plotted against Ct values. Lines: Ct = 40, Ct = 30, Ct = 25, and Ct = 20—blue; LOD for N protein concentration (1.00 ng/mL)—red. (**A**) all rtRT-PCR-positive samples with Triton X-100 and (**B**) without Triton X-100; (**C**) rtRT-PCR-positive samples mostly containing Wuhan-similar variants with Triton X-100 and (**D**) without Triton X-100; (**E**) rtRT-PCR-positive samples mostly containing Wuhan-distant variants with Triton X-100 and (**F**) without Triton X-100 t.

Diagnostic validation was performed in samples with and without Triton X-100 treatment (Table 3). The distribution of N protein concentration in samples plotted against Ct values obtained using the reference rtRT-PCR method is presented in Figure 4A,B.

From Table 3, it can be observed that the developed assay has low sensitivity, which is slightly higher in samples with Triton X-100 t (52.94% vs. 44.12%). The assay specificity is 100%, with a narrow 95% confidence interval, due to the absence of false positives (Figure 4A,B). The obtained PPV is high (100%) for both samples due to the absence of false positives. On the other hand, NPV is relatively low (about 70%) due to a high number of false negatives. The sample treatment with Triton X-100 slightly improves all tested parameters and, thus, ELISA performance. The obtained results suggest that the developed ELISA has low sensitivity and NPV; meanwhile, validated specificity and PPV are high. Relatively low ELISA test accuracy (75%), with a wider 95% CI, is the consequence of low true positives (Figure 4A,B) due to the ELISA’s low sensitivity.

As low sensitivity was obtained for positive samples with Ct < 40, in samples with and without Triton X-100, we have also evaluated the sensitivity obtained for rtRT-PCR-positive cases subcategorized according to Ct values (Table 4).

With the decrease in Ct cut-off from Ct < 40 to Ct < 30, ELISA sensitivity increased, and the highest sensitivity was obtained with Ct < 30 for samples with and without Triton X-100. At lower Ct values of rtRT-PCR-positive cases, ELISA sensitivity should be higher, as samples with lower Ct values have higher RNA titer and, thus, a higher level of N protein is expected in these samples. However, we obtained a bell-shaped curve from the dependence of ELISA sensitivity on Ct cut-off, where, with an increase in RNA level, sensitivity firstly increases to Ct < 30 and then decreases (Section S3, Figure S4). This decrease is the consequence of a higher decrease in true positives than of a decrease in false negatives (Figure 4A,B). These results suggest that even with a lower Ct cut-off (Ct < 30), ELISA sensitivity was still low and had a wide confidence interval (with Triton X-100 57.69% (CI 95% 36.92–76.65%)). This implies that the detected N protein level is highly underestimated, particularly for the samples containing the highest RNA titer (Ct < 20).

Obtained results implied that low sensitivity also could be related to the recognition of N protein from different SARS-CoV-2 variants. Antibodies used in the ELISA were generated against N protein that was produced based on a sequence of the original Wuhan variant, and, thus, our ELISA could be less sensitive to N protein from highly mutated variants. Therefore, the sensitivity of positive samples was subcategorized according to the variant similarity to the Wuhan SARS-CoV-2 variant, and assay sensitivity was recalculated. Samples positive for SARS-CoV-2 in rtRT-PCR testing were categorized into two groups: (a) the samples collected during 2020 and 2021, when the Wuhan, Alpha, and Delta variants were dominant, as samples mostly containing N protein from Wuhan and similar variants, and (b) the samples of patients collected during 2022, when the Omicron variant was dominant, i.e., the samples mostly containing N protein from Wuhan-distant variants. In parallel, ELISA sensitivity was evaluated for positive cases subcategorized according to Ct values, and the results are presented in Table 5. The distribution of N protein concentration in samples plotted against Ct values obtained using the reference rtRT-PCR method is presented in Figure 4C–F.

In comparison to the evaluation of all samples, the sensitivity of the ELISA for samples mostly containing Wuhan-similar variants was higher at all Ct cut-off values, with and without Triton X-100, and it is dramatically higher at lower Ct cut-off with and without Triton X-100 (Table 5). Moreover, it can be observed that, when the sensitivity of samples with mostly Wuhan-similar and distant variants was evaluated separately, the dependence of sensitivity on Ct cut-off is not bell-shaped but rather has an inverse correlation (Section S3, Figure S4). This means that sensitivity increases with RNA titer in samples, e.g., sensitivity increases with the N protein level in samples. For the samples with Wuhan-similar variants, this sensitivity increase is pronounced, reaching 100% at Ct < 25. However, this sensitivity increase for the samples with Wuhan-distant variants is negligible, suggesting that the recognition of N protein from Wuhan-distant variants by antibodies made against the Wuhan variant is poor despite their high RNA and N protein titer. The distribution of N protein concentration in samples and Ct values (Figure 4C,D) shows that the higher sensitivity for samples mostly containing Wuhan-similar variants than for all samples is due to the reduced number of false negatives originating from samples with Wuhan-distant variants. At Ct < 30, for the samples mostly containing Wuhan-similar variants, with and without Triton X-100 t, there is a much lower number of false negatives than at Ct < 40 with the same number of true positives (Figure 4C,D), resulting in pronouncedly higher sensitivity (75% vs. 50%). At Ct < 25, as there are no false negatives, sensitivity reaches 100%. On the other hand, there is dramatically lower sensitivity for the samples mostly containing Wuhan-distant variants in comparison to the evaluation of all samples at all Ct cut-off values (Table 5, Figure 4E,F). The reason for this is that about half of the false negatives obtained for the evaluation of all samples originate from samples with the Wuhan-distant variant.

Sample treatment with Triton X-100 slightly improved the performances of ELISA (Table 5). Although the Triton X-100 treatment does not increase assay sensitivity for all samples (Section S3, Figure S5), it enables three samples with very low N protein concentration to be above LOD, resulting in better parameters of diagnostic validation. Triton X-100 may decrease N protein masking using immunoglobulins by separating potential antigen–antibody complexes, thus allowing better N protein recognition through capture antibodies.

## 3. Discussion

Sensitive and accurate diagnostic methods are crucial for controlling infections and treating SARS-CoV-2. In this study, we have developed an ELISA that can detect the N protein of SARS-CoV-2. The assay was carried out with polyclonal nucleocapsid-specific antibodies from mice and rabbits that have been immunized with produced recombinant SARS-CoV-2 nucleocapsid protein. Analytical validation of the ELISA demonstrated that the assay has an LOD of 0.93 ng/mL and an LOQ of 5.3 ng/mL, where linear sensitivity is in the range of 1.52–48.83 ng/mL. The precision of the developed ELISA, evaluated using inter-day and intra-day assay variations, demonstrated that our ELISA has good precision in a broad concentration range covering more than three orders of magnitude. ELISA test accuracy, validated based on the recovery of N protein determined after the spiking of three simulated biological matrices with three spiking levels of N protein, correlated with the spiking dose. The CV% depended on matrix complexity, and it was the lowest for simulated serum (6% HSA), higher for simulated salivary fluid, and the highest for artificial urine, which was the most complex of all tested matrices. Pretreatment of N protein solution using heat at 56 °C for 1 h, with and without the addition of Triton X-100, had detrimental effects on analytical ELISA sensitivity. In contrast, N protein pretreatment with 1% TritonX-100 increased the analytical sensitivity of ELISA, and, therefore, ELISA diagnostic validation was performed with samples pretreated with and without 1% TritonX-100.

Diagnostic validation of the ELISA demonstrated that the assay has low sensitivity. Even with samples containing mostly Wuhan-similar variants at Ct < 40, the developed N protein ELISA satisfies neither the minimum requirements for ≥90% sensitivity defined by the European Commission for a diagnostic test (https://eur-lex.europa.eu/legal-content/EN/TXT/HTML/?uri=CELEX:32021H0122(01)&rid=1) (accessed on 1 September 2023) nor WHO recommendations, wherein the antigen test for diagnostic use should have a minimal sensitivity of ≥80% compared to the nucleic acid amplification test (NAAT) (https://apps.who.int/iris/bitstream/handle/10665/345948/WHO-2019-nCoV-Antigen-Detection-2021.1-eng.pdf?sequence=1&isAllowed=y) (accessed on 1 September 2023). However, the developed ELISA, with a specificity 100% (95% CI 91.19–100%), satisfies the ≥97% specificity required by both European Commission and WHO recommendations.

The discrepancy arises from the diminished analytical sensitivity of ELISA when contrasted with rtRT-PCR. Specifically, in samples characterized by low RNA titers, our ELISA failed to detect correspondingly low levels of N protein. Pollock et al. [13] provided insight into the performance of different antigen tests, showing that conventional N protein antigen tests, with LODs of 204, 121, and 2 pg/mL have a sensitivity of 47, 51, and 68%, respectively, while ultra-sensitive tests with an LOD of 0.16 pg/mL have 100%, in comparison to rtRT-PCR with Ct < 35. Although having one order of magnitude higher LOD than one of the assays in this study (1 ng/mL vs. 121 pg/mL), our ELISA has similar sensitivity (52.94% vs. 51%) in comparison to rtRT-PCR with Ct < 35 when all samples were tested. Similarly, Humbert et al. [9] obtained 58.6% sensitivity for N protein ELISA with an LOD of 8.4 pg/mL. This suggests that, despite such low analytical sensitivity (high LOD) and low absolute diagnostic sensitivity, our ELISA has relatively high diagnostic sensitivity concerning its analytical sensitivity. This can be explained by the usage of polyclonal antibodies as both capture and detection antibodies, which are produced in two different animals (rabbits and mice), thus recognizing a wide range of multiple N protein epitopes. As both mentioned studies were published in 2021, and their sample collection was conducted before the Omicron appearance, their studies were performed with samples containing Wuhan-similar variants, and, thus, the low recognition of N protein from Wuhan-distant variants can be excluded as the reason for the low sensitivity of antigen assays in these studies.

Wuhan’s similar sample group at Ct < 25 sensitivity reaches even 100% (Table 5); although, with a wide 95% CI due to the low number of samples, only four were obtained (Figure 4C,D). All four samples with Ct < 25 have the highest N protein level measured using our ELISA (29.9–40.8 ng/mL). This implies that, at a high viral load of Wuhan, similar variants in our assay have high diagnostic sensitivity. The obtained results confirm that our low ELISA diagnostic sensitivity is the consequence of both the poor recognition of N protein from Wuhan-distant variants by antibodies generated against recombinant Wuhan N protein, as well as low assay analytical sensitivity. The low assay analytical sensitivity is the consequence of the usage of polyclonal antibodies, as both capture and detection antibodies, in comparison to commercial sandwich ELISAs which contain at least one monoclonal antibody. In addition, antibodies used in our assay were purified using only protein A affinity chromatography. Therefore, although the used antibodies were highly purified, N protein-specific antibodies were not purified (using N protein-specific affinity purification), resulting in low ELISA performance. In the mentioned study of Humbert et al., [9] polyclonal antibodies raised only in rabbits were used as capture and detection antibodies. They obtained 100 times higher analytical sensitivity than in our study (8.4 pg/mL vs. 1 ng/mL) due to affinity-purification against N protein. However, by using polyclonal antibodies from two species (rabbits and mice), providing a wider repertoire of antibodies against N protein epitopes, we obtained similar diagnostic sensitivity as in this study (62.50% with and 58.33% without Triton X-100 vs. 58.6%). In addition, to more effectively capture antigen epitopes, in this study, rabbit antibodies were used as capture antibodies since, in general, in comparison to mice, rabbit antiserum consists of a larger variety of antibodies, recognize more epitopes per protein antigen since there is less immunodominance in rabbit, and, thus, have a better affinity and specificity.

Several mutations have occurred in the N protein from the Omicron variant, resulting in conformational changes that affect the binding of specific antibodies in the antigen tests. At the beginning of 2022, the warning was raised on the possibility that the detection of Omicron variants by using available reagents/kits would not be efficient for the detection of previous variants [14]. The recent systematic review and meta-analysis of the clinical performance of rapid antigen tests (RATs) in comparison to RT-PCR for SARS-CoV-2 detection in the Omicron variant reported that RATs show impaired performance for COVID-19 diagnoses when the Omicron variant is circulating, particularly in samples with low viral loads. Moreover, for the detection of the Omicron variant, the pooled sensitivity of WHO-approved and FDA-approved kits, separately, was calculated to be 68% (95% CI 59.4–75.6%) and 72.8% (95% CI 62.0–81.5%), respectively, suggesting that even WHO-approved kits did not meet the appropriate standards for the detection of the Omicron variant. These data confirm the results obtained in this study, e.g., N protein antigen tests based on antibodies against the Wuhan variant are much less sensitive for the detection of N protein from Wuhan-distant variants such as Omicron. All rtRT-PCR negatives were also negative in ELISA (there were no false positives), resulting in a very narrow 95% CI, thus ruling out the possibility of detecting the N proteins of other coronaviruses. On the other hand, a decrease in sensitivity from 60% for Wuhan-similar variants to 30% with Wuhan-distant variants is due to poorer recognition of N protein from Wuhan-distant variants. N protein sequence homology between different variants of the same strain of SARS-CoV-2 is only slightly less than 100% due to several mutations, while sequence homology between the N protein of different strains of coronaviruses is much lower. For example, sequence homology between SARS-CoV-2 and SARS-CoV-1 is only 89.1% [4]. Therefore, it is expected that the sensitivity of our ELISA for other coronaviruses, e.g., for different strains of coronaviruses, is extremely low due to very poor recognition of the N protein of other coronaviruses via antibodies against N protein from Wuhan SARS-CoV-2, resulting in such high ELISA specificity.

Although many antigen assays have been made available for the detection of SARS-CoV-2 antigens, their relatively high cost, particularly during the first year of epidemics, prevented many laboratories, especially in developing countries, from their broad use. Expression in *E. coli* and the purification of viral antigens using immobilized metal affinity chromatography (IMAC) is cost-effective. In comparison to the production of monoclonal antibodies, the production of polyclonal antibodies against produced recombinant viral antigens is not only fast and inexpensive, but it is also less demanding, requiring less technical skill and specialized equipment, with fewer production challenges. Moreover, the biophysical diversity of polyclonal antibodies enables their greater stability to environmental challenges, allowing their easier storage and handling [15]. Therefore, the production of the most expensive components of antigen assays in cost-efficient processes, as presented in this study, would result in more affordable antigen tests. Moreover, the total working time for our developed N protein ELISA test is about 5–6 h, which is similar to the turnaround time for rtRT-PCR, which is about 4–6 h [16].

## 4. Materials and Methods

### 4.1. Material and Reagents

Unless otherwise stated, all chemicals were purchased from Sigma-Aldrich (St. Louis, MO, USA). All chemicals were analytical reagent grade, and Milli-Q water (18 MΩ·cm at 25 °C) (Millipore, Bedford, MA, USA) was used for all experiments.

### 4.2. Production and Characterization of Nucleocapsid Protein

Cloning and the expression of the N protein recombinant fragment (rfNP, 58-419 aa) were performed exactly as described by Djukic et al. [17]. Purified rfNP was stored at −20 °C in 20 mM phosphate buffer containing 320 mM NaCl and 5% glycerol. The concentration of N protein was determined spectrophotometrically at 280 nm. The extinction coefficient for proteins under native conditions (ε) was calculated from the equation proposed by Pace et al. [18] ε(mL mg^−1^ cm^−1^) = (5500 nW + 1490 nY + 125 nC), where nW, nY, and nC are the numbers of Trp, Tyr, and Cys, respectively, per polypeptide chain, and M is the molecular mass (Da). For rfNP, M = 40,100 Da, nW = 4, nY = 11, and nC = 0, from which calculated ε = 38,390 mL mg^−1^ cm^−1^. Purified rfNP (in further text N protein) was characterized using high-resolution mass spectrometry. Briefly, after SDS–PAGE gel protein bands were in-gel digested using trypsin, followed by nLC-ESI-MS/MS analysis, the identification of proteins and post-translational modification (PTM) profiling was performed using PEAKS Suite X (Bioinformatics Solutions Inc., Waterloo, ON, Canada; Section S4 in Supplementary Material).

### 4.3. Sera Production and Testing of Reactivity of Polyclonal Antibodies toward N Protein

#### 4.3.1. Antisera Production

The antibodies against N protein were raised in rabbits and mice according to Harboe and Ingild [19]. Mice (n = 10) were immunized with rfNP formulated with CFA (first dose, 100 µg N protein/mice) or IFA (second and third doses, 50 µg N protein/mice) in three-week intervals. Rabbits (n = 2) were immunized with N protein formulated with CFA (first dose) or IFA (second and third doses) subcutaneously in four sites (1 mg N protein/rabbit) in two-week intervals. The first bleeding of mice was performed two weeks after the third dose, and, then, in intervals not shorter than two weeks. The first bleeding of rabbits was performed two weeks after the second dose, and the final blood collection was performed three weeks after the third dose. Sera collected from the same animal species were pooled and used for further experiments. Detailed immunization schemes for both animal species are presented in Section S5.1.

Polyclonal antibodies were purified from collected rabbits and mice sera pools through ammonium-sulfate (AS) precipitation. The AS-purified, high-titer rabbit sera antibodies were further purified using protein A affinity chromatography. The obtained highly purified rabbit antibodies were used as capture antibodies in the ELISA. Polyclonal antibodies purified from mice sera using AS precipitation were used as detection antibodies in the ELISA. Detailed protocols for the purification of antibodies from animal sera are given in the supplementary section (Section S5.2).

#### 4.3.2. Checking of Reactivity of Polyclonal Antibodies toward N Protein Using Indirect ELISA and Western Blot

Indirect ELISA: Before and after each purification step, the reactivity of polyclonal antibodies toward N protein was checked using an indirect ELISA. To determine the working dilution of polyclonal antibodies/sera, wells were coated with N protein, and the serial dilution of polyclonal antibodies/sera pools was tested. Briefly, ELISA plates (Nunc, Maxisorp, Roskilde, Denmark) were coated with N protein (5 μg/mL in 50 mM carbonate buffer pH 9.5–9.6), 100 µL per well, overnight at 4 °C in a humid atmosphere. The plates were rinsed 3 times with Tris-buffered saline with 0.05% Tween (TTBS, 25 mM Tris, 0.15 M NaCl, 0.05% Tween-20, pH 7.5). The plates were blocked via incubation with 1% bovine serum albumin (BSA) in TTBS, 300 µL per well, for 1 h, at room temperature (RT). Double serial dilutions of rabbit and mice sera, from 1:250 to 1:256.000 in 1% BSA in TTBS, were prepared. Dilution 1:250 was prepared in vial tubes in duplicate, and serial dilutions were made in another standard microtiter plate. Serial sera dilutions were added into blocked and rinsed plates (100 µL per well) and incubated for 1 h at RT. Detection antibodies were diluted in 1% BSA/TTBS. Goat anti-rabbit antibody conjugated with alkaline phosphatase (GαR-AP, Bio-Rad AbD Serotec GmbH, Hercules, CA, USA) was diluted 1:5000, and goat anti-mouse antibody conjugated with alkaline phosphatase (GαM-AP, Sigma -Aldrich (St. Louis, MO, USA) was diluted 1:15000. The plates were incubated with detection antibodies, 100 µL per well, for 1 h, at RT. AP substrate (1 mg/mL para-nitrophenylphosphate in 10 mM diethanolamine buffer with 0.5 M MgCl_2_ pH 9.6) was prepared. The plates were incubated with AP substrate for up to 1 h at room temperature. The absorbance at 405 nm was recorded using an ELISA plate reader (BioTek Instruments, Winooski, VT, USA), and the mean values of the two replicas were presented.

**Western blot:** The reactivity of obtained purified mice and rabbit antibodies against N protein was investigated using Western blot. Following SDS–PAGE on 12% polyacrylamide gel, proteins were transferred to a nitrocellulose membrane using an electroblotting system (VWR; Darmstadt, Germany). The membrane was probed with either the rabbit or mice serum diluted 5000 times in 0.1% BSA in Tris-buffered saline containing Tween-20 (TBST; 20 mM Tris-buffer, 0.9% NaCl, 0.2% Tween-20; pH 7.4). The bound IgG was detected using alkaline phosphatase-labeled goat anti-rabbit IgG antibodies (Sigma–Aldrich, St. Louis, MO, United States; 15,000 times diluted in TBST containing 0.1% BSA) or biotinylated goat anti-mouse IgG antibodies (Sigma–Aldrich, St. Louis, MO, USA; 200,000 times diluted in TBST containing 0.1% BSA), followed by incubation with streptavidin conjugated with alkaline phosphatase (Roche; Basel, Switzerland; 1000 times diluted in TBST containing 0.1% BSA). Control samples containing no primary antibodies were included. Immunoblot was visualized with a substrate solution containing 1.5 mg 5-bromo-4-chloro-3-indolyl phosphate and 3 mg nitroblue tetrazolium in 10 mL 100 mM carbonate-bicarbonate buffer containing 5 mM MgCl_2_, pH 9.5.

### 4.4. Sandwich ELISA for Quantification of SARS-CoV-2 N Protein

The 96-well plates (NUNC Maxisorp, Thermo Fisher Scientific; Roskilde, Denmark) were coated with 100 μL per well of rabbit polyclonal antibodies, purified using protein A-affinity chromatography, at a concentration of 5 µg/mL in coating buffer (50 mM carbonate buffer pH 9.5–9.6), and left overnight at 4 °C. Plates were washed five times with 300 μL per well of wash buffer (TTBS, pH 7.4). After washing, wells were blocked with 300 μL of blocking buffer (1% BSA in TTBS) for 1 h at RT. For the calibration curve, N protein standards were prepared as double serial dilutions (starting from 400 to 0.78 ng/mL) in the blocking buffer. After blocking, 100 μL of standards (in duplicate) and samples (in duplicate) were pipetted into designated wells along with four blank samples (1% BSA in TTBS) and incubated for 1 h at RT. Plates were then washed five times with TTBS and incubated with detection mouse polyclonal antibodies (diluted 1:1250 in blocking buffer), 100 μL per well, for 1 h at RT. Plates were washed five times with TTBS and incubated with secondary antibodies (goat anti-mouse biotin, diluted 1:100,000 in blocking buffer, Sigma–Aldrich), 100 μL per well, and incubated for 1 h at RT. Plates were washed five times with TTBS and incubated with streptavidin–alkaline phosphatase (AP) conjugate solution (1:5000 dilution in blocking buffer, Sigma–Aldrich, St. Louis, MO, USA), 100 μL per well, for 1 h at RT. Finally, plates were washed three times with TTBS and twice with TBS (Tris-buffered saline, pH 7.4) before incubating with freshly prepared AP substrate (1 mg/mL para-nitrophenyl phosphate in 10 mM diethanolamine buffer with 0.5 M MgCl2 pH 9.6), 100 μL per well, at 37 °C. The absorbance at 405 nm was recorded using an ELISA plate reader (Pharmacia LKB, Uppsala, Sweden) after 1 h of incubation.

### 4.5. Analytical Validation of Capture ELISA for Quantification of SARS-CoV-2 N Protein

#### 4.5.1. Sensitivity and Linear Range

The average Limit of Detection (LOD) was expressed as the concentration calculated from the curve of the sum of the mean value of the absorbances corresponding to the blank samples and the standard deviation of the absorbances corresponding to the blank samples multiplied by 3 (LOD = mean Cblank + 3 SDblank). The average Limit of Quantification (LOQ) was expressed as the concentration calculated from the curve of the sum of the mean value of the absorbances corresponding to the blank samples and the standard deviation of the absorbances corresponding to the blank samples multiplied by 10 (LOQ = mean Cblank + 10 SDblank) [20].

#### 4.5.2. Intra-Day and Inter-Day Variability

The ELISA prototype’s intra-day variability was validated by running the N protein standard (double serial dilutions starting from 400 ng/mL to 0.78 ng/mL) in duplicate, along with four blank samples, in three independent experiments, while inter-day variability was validated in the same way in three independent experiments in three days. The same person conducted the ELISA for the testing of intra-day and inter-day variability. Intra-day and inter-day precision was expressed as the coefficient of variation (CV%).

#### 4.5.3. N Protein Recovery in Different Simulated Biological Fluids

To validate the ELISA prototype’s ability to quantify N protein in biological fluids, 6% HSA (Human Serum Albumin) in phosphate-buffered saline (PBS) was used as a serum substitute [21], as well as simulated salivary fluid (SSF) [22] and artificial urine (AU) [23]. Compositions of simulated body fluids are provided in Section S6 (Tables S2–S4). Simulated body fluids were spiked with recombinant N protein to achieve the final concentrations of 6, 24, and 96 ng/mL in the blocking buffer (in duplicate). Non-spiked simulated body fluids with the blocking buffer were used as control samples. The recovery of the N protein was expressed as a percentage of the determined N protein concentration in the simulated biological fluids of the spiked concentration of N protein in the samples.

### 4.6. Clinical Samples Collection

Developing the N protein-targeting sandwich ELISA involved testing of nasopharyngeal swabs that were received at the Institute of Virology, Vaccines, and Sera–Torlak for rtRT-PCR testing to SARS-CoV-2 in the scope of the standard diagnostic procedure of COVID-19. Nasopharyngeal swabs were immersed in 1 mL of the viral transport medium (VTM). After analysis using rtRT-PCR, the remains were used for the N protein ELISA.

The swabs were analyzed for the presence of SARS-CoV-2 by using the Novel Coronavirus (2019-nCoV) Nucleic Acid Diagnostic Kit (Sansure Biotech Inc., Changsha, China) in accordance with the manufacturer’s instructions. The kit allows the detection of ORF1ab and N genes of SARS-CoV-2. Swabs that were positive for the presence of SARS-CoV-2 (Ct (N) < 40, Ct (ORF1ab) < 40) were collected during the following periods: April 2020 (n = 2, with confirmed Wuhan variant), November–December 2020 (n = 20, alpha variant dominant in circulation, not confirmed variants), October–November 2021 (n = 2, with confirmed delta variant), and September 2022 (n = 10, Omicron variant dominant in circulation, not confirmed variants) [24]. Swabs that were shown by rtRT-PCR as SARS-CoV-2 negative ((Ct (N) > 40, Ct (ORF1ab) > 40, Ct (internal control) < 35) were also included in ELISA testing and collected during November–December 2020 (n = 26) and September 2022 (n = 14). Collected swabs were stored at −80 °C until analysis. Both processing and subsequent testing were performed with operators blinded to infection status and Ct value.

#### Sample Inactivation Protocols

***Thermal inactivation***: The samples in VTM were mixed with blocking buffer (1% BSA in Tris-buffered saline with 0.01% Tween 20) at a ratio of 1:1, *v*/*v*, and incubated for 1 h at 56 °C.

***Chemical inactivation***: The samples in VTM were mixed with a 2% Triton X-100 solution in a blocking buffer in a 1:1 (*v*/*v*) ratio, so that the final Triton X-100 concentration in the samples was 1%, and incubated for 1 h at RT.

***Combination of chemical and thermal inactivation***: The samples in VTM were mixed with a 2% Triton X-100 solution in a blocking buffer in a 1:1 (*v*/*v*) ratio followed by incubation for 1 h at 56 °C.

***No-treatment***: The samples in VTM were mixed with a blocking buffer (1:1, *v*/*v*) and incubated for 1 h at RT.

### 4.7. Ethics

The animals were treated according to Directive 2010/63/EU of the European Parliament and the Council of 22 September 2010 on the protection of animals used for scientific purposes. Immunization of rabbits for the production of N protein targeting serum was approved by the Ethical Committee for the Use of Laboratory Animals of the Institute for the Application of Nuclear Energy “INEP” and the Decision on approval to conduct animal testing at the INEP Institute for the Application for Nuclear Energy (Case No. 323-07-10579/2021-05, November 2021) was issued by the Ministry of Agriculture, Forestry and Water Management of the Republic of Serbia—Veterinary Administration regarding rabbit immunization.

The immunization of mice for the production of the N protein targeting serum was approved by the Ethics Committee for the Welfare of Experimental Animals at the Institute of Virology, Vaccines, and Sera–Torlak (approval for request No. 1/22).

As human sample residual clinical swab samples were used in the study, the use of swabs in this study was approved by the Ethics Committee of the Institute of Virology, Vaccines, and Sera–Torlak (approval for request No. 490/1). Written informed consent for participation was not required for this study following the national legislation and the institutional requirements.

### 4.8. Statistical Analyzes

The data are presented as the mean ± S.D. Unless otherwise stated, all experiments were conducted twice. Data were analyzed using one-way ANOVA with Tukey’s multiple comparison test at a significance level of 0.05. The diagnostic sensitivity was calculated as the proportion of the true SARS-CoV-2-positive samples of rtRT-PCR-positive samples.

The diagnostic specificity was calculated as the proportion of the true SARS-CoV-2-negative samples of total rtRT-PCR-negative samples. Positive predictive value (PPV) was calculated as the proportion of the true SARS-CoV-2-positive samples of all ELISA-positive samples, while the negative predictive value (NPV) was calculated as the proportion of the true SARS-CoV-2-negative samples of all ELISA-negative samples. Accuracy was calculated as the proportion of the true SARS-CoV-2-positive and true SARS-CoV-2 -negative samples of the total samples. The results were presented with 95% CI to assess the level of uncertainty introduced by sample size calculated using the Wilson’s score method.

## 5. Conclusions

The obtained results suggest that developed N protein ELISA sensitivity could be improved by increasing its analytical sensitivity, e.g., by using mice and rabbit N-specific antibodies obtained using affinity purification against N protein. However, even then, it seems that, for Wuhan’s distant variants, such as Omicron and its descendants, it cannot reach sensitivity that meets WHO and European Commission recommendations. On the other hand, our results imply that using polyclonal antibodies from two different species and enabling the recognition of a wide range of multiple N protein epitopes can provide high sensitivity even with ELISAs with lower analytical sensitivity. The ELISA developed in this study is a fast, simple, and affordable approach for antigen testing.

## Figures and Tables

**Table 1:** Intra-day precision (N = 2) and inter-day precision (N = 2) of the N protein ELISA expressed as a percentage of CV.

N Protein Conc. (ng/mL)	Intra-Day Precision (CV%)	Inter-Day Precision (CV%)
400	1.51	14.41
200	4.57	13.14
100	3.73	15.06
50	4.1	13.37
25	6.61	11.33
12.5	4.87	16.35
6.25	5.79	18.83
3.12	9.13	20.21
1.56	16.48	35.72
0.78	20.69	46.03

**Table 2:** Recovery of N protein in simulated biological fluids using the ELISA prototype.

Simulated Biological Fluid	Spiked Con. (ng/mL)	Measured Con. (ng/mL)	Recovery (%)	CV (%)
Simulated Salivary Fluid (SSF)	100	81.37	81.37 ± 0.68	0.83
	25	11.77	48.20 ± 28.00	58.09
	6.25	3.55	58.22 ± 6.80	11.68
6% Human Serum Albumin (HSA)	100	96.49	96.49 ± 17.16	17.78
	25	17.16	68.63 ± 12.40	18.07
	6.25	3.86	61.70 ± 8.96	14.52
Artificial Urine (AU)	100	105.72	105.72 ± 50.80	48.05
	25	15.67	62.67 ± 44.91	71.66
	6.25	2.48	39.70 ± 10.22	25.74

**Table 3:** The number of true positive, false positive, true negative, and false negative samples and parameters of the clinical validation of N protein ELISA using rtRT-PCR (Ct < 40) in samples with and without Triton X-100 treatment.

Samples with Triton X-100				
		rtRT-PCR (Ct ≤ 40)		
		Positive	Negative	Sum
N protein ELISA	Positive	18	0	18
	Negative	16	40	56
Sum		34	40	74
Sensitivity (%) (95% CI)	% Specificity (95% CI)	% Accuracy (95% CI)	PPV (positive predictive value) (95% CI)	NPV (negative predictive value) (95% CI)
52.94% (35.13–70.22%)	100% (91.19–100.00%)	78.38% (67.28–87.11%)	100.00% (81.47–100.00%)	71.43% (63.64–78.12%)
Samples without Triton X-100				
		RT-PCR (Ct ≤40)		
		Positive	Negative	Sum
N protein ELISA	Positive	15	0	15
	Negative	19	40	59
Sum		34	40	74
% Sensitivity (95% CI)	% Specificity (95% CI)	% Accuracy (95% CI)	PPV (positive predictive value) (95% CI)	NPV (negative predictive value) (95% CI)
44.12% (27.19–62.11%)	100% (91.19–100.00%)	74.32% (62.84–83.78%)	100.00% (78.20–100.00%)	67.80% (60.96–73.94%)

**Table 4:** N protein ELISA diagnostic sensitivity dependence for rtRT-PCR-positive cases subcategorized according to Ct values.

Positive RT-PCR	Sensitivity (%) (95% CI)	
	With Triton X-100	Without Triton X-100
Ct < 20, n = 11	46.15 (19.22–74.87%)	36.36% (10.93–69.21%)
Ct < 25, n = 13	53.85% (25.13–80.78%)	38.46% (13.86–68.42%)
Ct < 27, n = 19	52.63% (28.86–75.55%)	42.11% (20.25–66.50%)
Ct < 30, n = 26	57.69% (36.92% to 76.65%)	50.00% (29.93–70.07%)
Ct < 35, n = 34	52.94% (35.13–70.22%)	44.12% (27.19–62.11%)
Ct < 40, n = 34	52.94% (35.13–70.22%)	44.12% (27.19–62.11%)

**Table 5:** N protein ELISA diagnostic sensitivity dependence for rtRT-PCR-positive cases subcategorized according to variant similarity to the Wuhan variant and Ct values.

**Positive RT-PCR**	**Sensitivity (%) (95% CI) for Samples Mostly Containing Wuhan-similar variants (Wuhan, Alpha, Delta), n = 24**	
	**With Triton X-100**	**Without Triton X-100**
Ct < 20, n = 3	100.00% (29.24–100.00%)	100.00% (29.24–100.00%)
Ct < 25, n = 4	100.00% (39.76–100.00%)	100.00% (39.76–100.00%)
Ct < 27, n = 10	70.00% (34.75–93.33%)	70.00% (34.75–93.33%)
Ct < 30, n = 16	75.00% (47.62% to 92.73%)	75.00% (47.62% to 92.73%)
Ct < 35, n = 24	62.50% (40.59% to 81.20%)	58.33% (36.64–77.89%)
Ct < 40, n = 24	62.50% (40.59% to 81.20%)	58.33% (36.64–77.89%)
**Positive RT-PCR**	**Sensitivity (%) (95% CI) for samples mostly containing Wuhan-distant variants (Omicron), n = 10**	
	**With Triton X-100**	**Without Triton X-100**
Ct < 20, n = 8	37.50% (8.52–75.51%)	12.50% (0.32% to 52.65%)
Ct < 25, n = 9	33.33% (7.49% to 70.07%)	11.11% (0.28% to 48.25%)
Ct < 27, n = 9	33.33% (7.49% to 70.07%)	11.11% (0.28% to 48.25%)
Ct < 30, n = 10	30.00% (6.67–65.25%)	10.00% (0.25% to 44.50%)
Ct < 35, n = 10	30.00% (6.67–65.25%)	10.00% (0.25% to 44.50%)
Ct < 40, n = 10	30.00% (6.67–65.25%)	10.00% (0.25% to 44.50%)

## Data Availability

The data that support the findings of this study are available from the corresponding author, T.C.V., upon reasonable request.

## Supplementary Materials

The following supporting information can be downloaded at: https://www.mdpi.com/article/10.3390/ijms25010333/s1. Reference [25] are cited in the supplementary materials.
