# Supplementary material for: Sandwich ELISA for the Quantification of Nucleocapsid Protein of SARS-CoV-2 Based on Polyclonal Antibodies from Two Different Species
Source: Int J Mol Sci. 2023 Dec 26;25(1):333. doi: 10.3390/ijms25010333 (PMC10778659; doi:10.3390/ijms25010333)
Supplement: Supplementary file 1 [file ijms-25-00333-s001.zip › ijms-2756843-supplementary.pdf]

Sandwich ELISA for quantification of Nucleocapsid Protein of SARS-COV-2 based on polyclonal antibodies

Maja Mladenovic Stokanic[1], Ana Simovic[1], Vesna Jovanovic[1], Mirjana Radomirovic[1], Bozidar Udovicki[2], Maja Krstic Ristivojevic[1], Teodora Djukic[5], Tamara Vasovic[1], Jelena Acimovic[1], Ljiljana Sabljic[3], Ivana Lukic[4], Ana Kovacevic[4], Danica Cujic[3], Marija Gnjatovic[3], Katarina Smiljanic[1], Marija Stojadinovic[1], Jelena Radosavljevic[1], Dragana Stanic-Vucinic[1], Marijana Stojanovic[6], Andreja Rajkovic [2,8] and Tanja Cirkovic Velickovic[1,7,8,9]

1 - University of Belgrade–Faculty of Chemistry, Department of Biochemistry, Centre of Excellence for Molecular Food Sciences, Studentski trg 12-16, 11000 Belgrade, Serbia

2 - Department of Food Safety and Quality Management, Faculty of Agriculture, University of Belgrade, Nemanjina 6, 11080, Zemun, Belgrade, Serbia

3 - University of Belgrade, Institute for the Application of Nuclear Energy – INEP, Banatska 31b, 11080 Zemun, Belgrade, Serbia

4 - Institute of Virology, Vaccines, and Sera–TORLAK, Vojvode Stepe 458, 11152 Belgrade, Serbia

5 - University of Belgrade, Faculty of Medicine, Institute of Medical Chemistry, Višegradska 26, 11000 Belgrade, Serbia

6 - Department of Molecular Biology, Institute for Biological Research "Siniša Stanković", University of Belgrade, 142 Despot Stefan Blvd., 11000, Belgrade, Serbia

7 - Serbian Academy of Sciences and Arts, Kneza Mihaila 35, 11000 Belgrade, Serbia

8 - Ghent University, Faculty of Bioscience Engineering, Ghent, Belgium

9-Ghent University Global Campus, Incheon, South Korea

I Results

S1. Identification of N protein by HRMS

S1.1. Checking of N protein purity

Recombinant N protein purity was checked after imidazole removal and buffer exchange by SDS PAGE (**Figure 6**.). For comparison, commercial high-purity HSA was also analyzed.

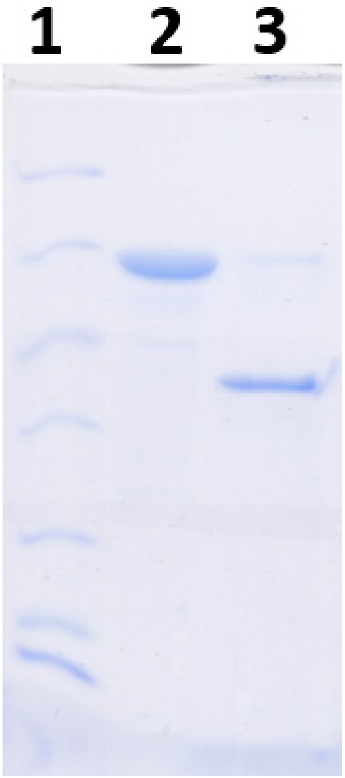

Figure S1. SDS PAGE of N protein purity double check after imidazole removal and buffer exchange: lane 1 - MW markers, lane 2- HSA, lane 3 – N protein produced on a small scale and purified by a combination of immobilized metal affinity chromatography (IMAC) and ion exchange chromatography (IEC).

S1.2. Identification of N protein

Tandem mass spectrometry identification of proteins in an in-gel digested band of N protein (Figure S1, lane 3), confirmed the identity of N protein with high scores and peptide coverage (Fig. S2.).

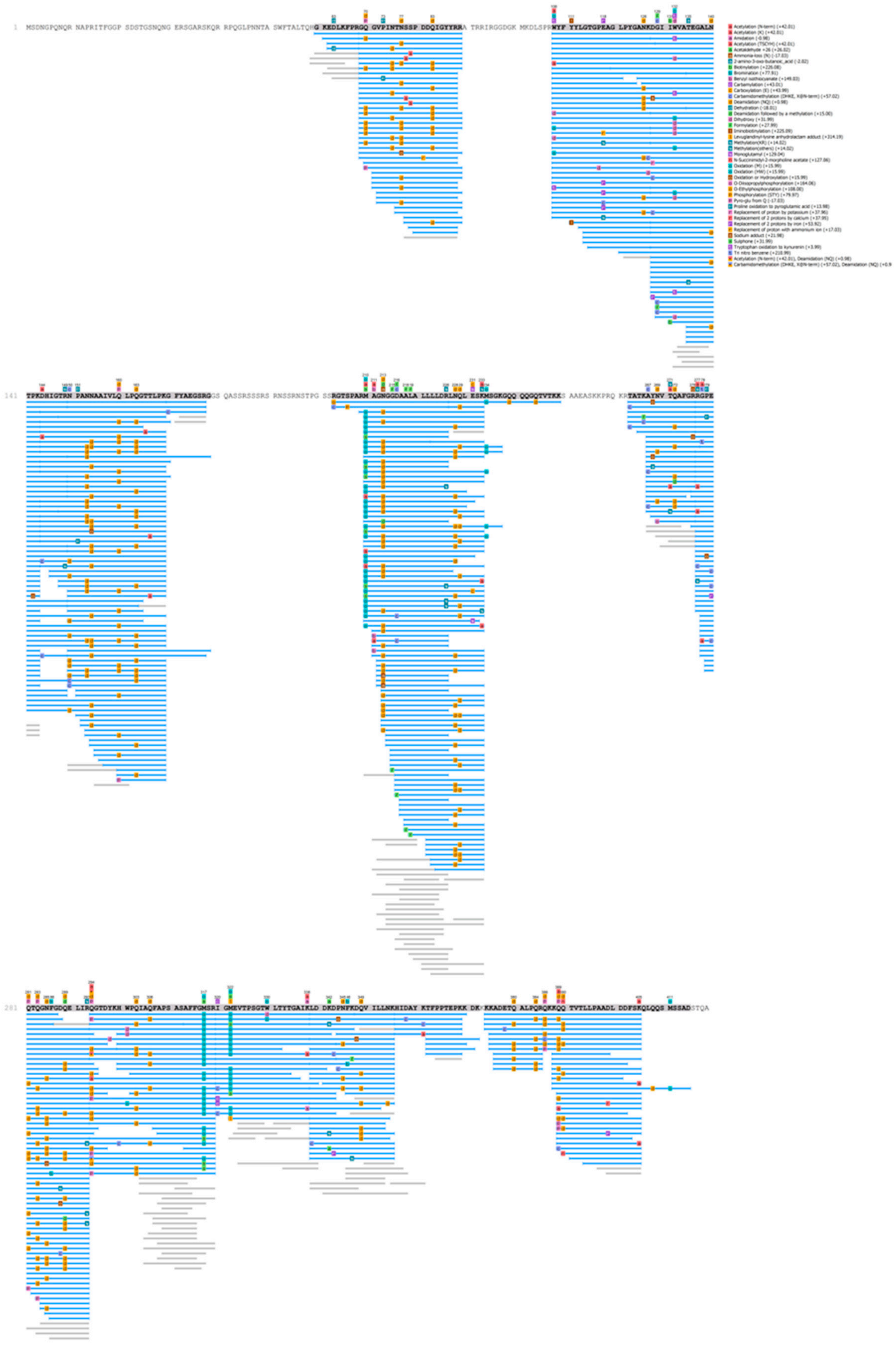

Acetylation (N-term) (+42.01)
Acetylation (K) (+42.01)
Amidation (-0.98)
Acetylation (TSCYH) (+42.01)
Acetaldehyde +26 (+26.02)
Ammonia-loss (N) (-17.03)
2-amino-3-oxo-butanoic_acid (-2.02)
Biotinylation (+226.08)
Bromination (+77.91)
Benzyl isothiocyanate (+149.03)
Carbamylation (+43.01)
Carboxylation (E) (+43.99)
Carbamidomethylation (DHKE, X@N-term) (+57.02)
Deamidation (NQ) (+0.98)
Dehydration (-18.01)
Deamidation followed by a methylation (+15.00)
Dihydroxy (+31.99)
Formylation (+27.99)
Iminobiotinylation (+225.09)
Levuglandinyl-lysine anhydrolactam adduct (+314.19)
Methylation(KR) (+14.02)
Methylation(others) (+14.02)
Monoglutamyl (+129.04)
N-Succinimidyl-2-morpholine acetate (+127.06)
Oxidation (M) (+15.99)
Oxidation (HW) (+15.99)
Oxidation or Hydroxylation (+15.99)
O-Diisopropylphosphorylation (+164.06)
O-Ethylphosphorylation (+108.00)
Phosphorylation (STY) (+79.97)
Pyro-glu from Q (-17.03)
Proline oxidation to pyroglutamic acid (+13.98)
Replacement of proton by potassium (+37.96)
Replacement of 2 protons by calcium (+37.95)
Replacement of 2 protons by iron (+53.92)
Replacement of proton with ammonium ion (+17.03)
Sodium adduct (+21.98)
Sulphone (+31.99)
Tryptophan oxidation to kynurenin (+3.99)
Tri nitro benzene (+210.99)
Acetylation (N-term) (+42.01), Deamidation (NQ) (+0.98)
Carbamidomethylation (DHKE, X@N-term) (+57.02), Deamidation (NQ) (+0.9

Figure S2. Sequence coverage of N protein, obtained after mass spectrometry analysis of purified recombinant N protein;

SARS-CoV-2 Nucleoprotein (UniProtKB entry P0DTC9 https://www.uniprot.org/uniprot/P0DTC9) was identified as the most intense and abundant protein fragment with PEAKS PTM score of >500 and sequence coverage of over 70%, including 183 unique peptides. The half of missing covered N protein sequences were within the central linker domain (Fig S2.).

Table S1. Identification of N protein from the band of purified recombinant N protein (Figure S1, lane 3) after its in-gel digestion and MS analysis.

Accession number / Protein Name	Score	Coverage (%)	Unique peptides
P0DTC9|NCAP_SARS2 Nucleoprotein OS=Severe acute respiratory syndrome coronavirus 2, 46 kDa	**504.9**	**74.22**	**183**

S2. Purification of polyclonal antibodies from mice and rabbit sera

For the development of an ELISA test specific for the detection of SARS-CoV-2 N protein, recombinantly produced N protein was used for the immunization of mice and rabbits. Sera obtained from rabbits and mice were then tested for titer and specificity (Figure S3 and Figure 1). To determine the titer of polyclonal sera required to detect N protein in samples, we use wells coated with N protein and serial dilution of sera pools from different animals. After multiple washing steps, we detected the binding of rabbit and mice antibodies using secondary biotinylated antibodies and streptavidin-alkaline phosphatase chimaera or secondary antibodies with previously coupled alkaline phosphatase, where the amount of enzymes' substrate converted to the product was measured as an increase in absorbance at 405 nm.

As shown in Figure S3A, unpurified sera pools from both animals showed very high titers and expected logarithmic decrease of signal with dilution. Based on the obtained data titer for unpurified sera was determined to be X. The same trend was observed for pools purified using AS precipitation and rabbit sera purified using protein A affinity chromatography (Figure S3B and S3C). As shown in Figure S3D, clear bands from antibodies could be observed in both full and purified samples. Western blot analysis showed only one protein band on mass around 40 kDa, a

mass of purified N protein suggesting that the obtained sera is highly specific for N protein (Figure 2).

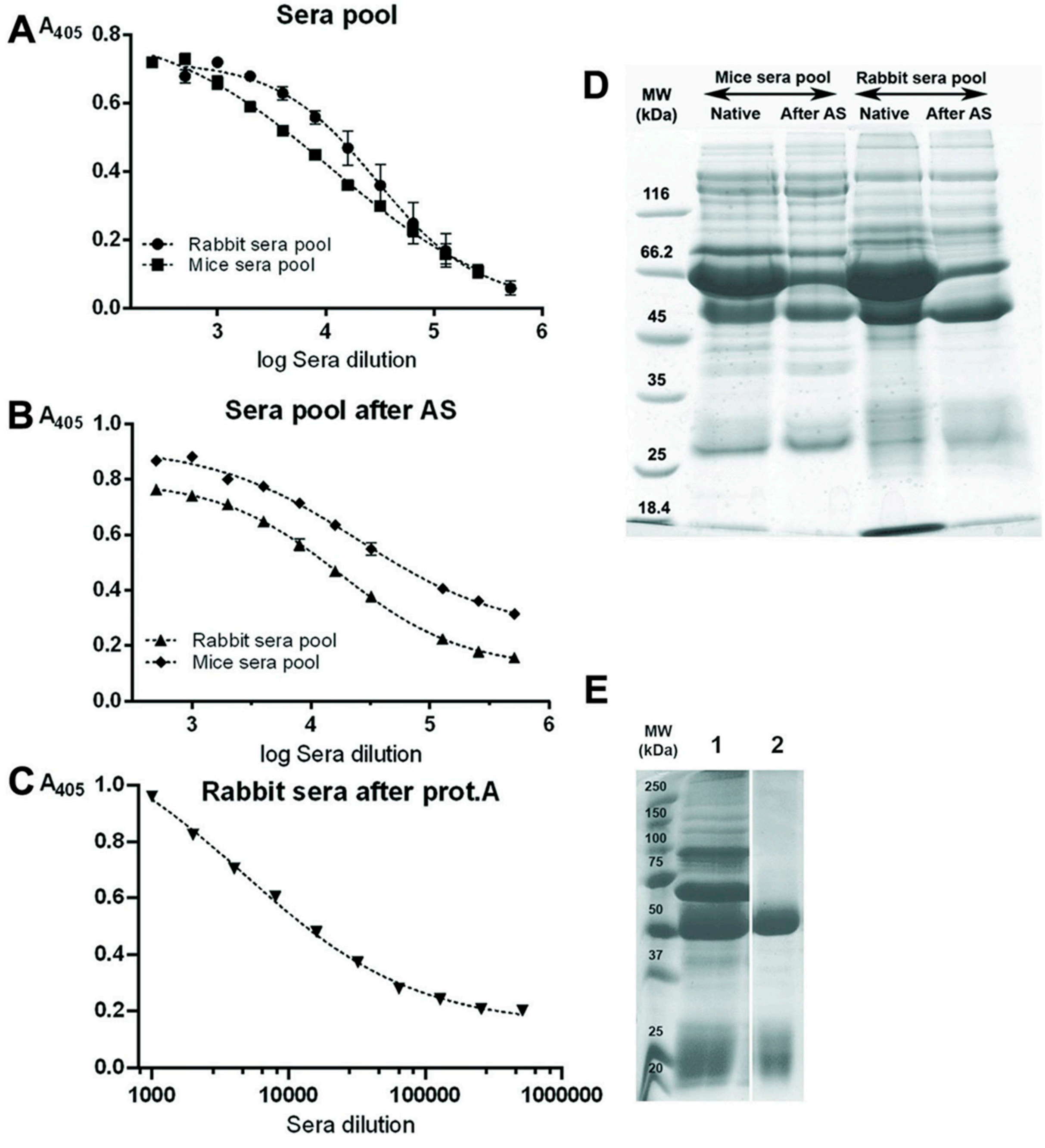

Figure S3. A. Direct ELISA for detection of IgG binding from obtained mice and rabbits sera pools after animals immunization by recombinant SARS-CoV-2 N protein to plate coupled with N protein (sera pools were obtained from 10 mice after two blood collections and from two rabbits after the first blood collection), B. After purification by AS precipitation, C. Direct ELISA for

detection of IgG binding from rabbit sera poll after purification by affinity chromatography on Protein A Sepharose, D. Protein profiles of mice and rabbit sera pools resolved by SDS-PAGE prior and after AS precipitation and E. Protein profile of rabbit sera pool resolved by SDS-PAGE 1-after AS precipitation and 2-after protein A-affinity chromatography.

Section S3 Diagnostic validation

S3.1. Stabilization of capture antibodies

Pre-coated ELISA plates were prepared for usage in clinical practice. To ensure the preservation of the biofunctionality of the surface-bound capture antibodies, the commonly used stabilizing excipient, 3% sucrose with 10% glycerol in MilliQ water was used. The plates were incubated with 300 μL per well of a stabilizing agent for 1 hour at room temperature. After an hour of incubation, the solution was carefully aspirated from each well. The plate was then blotted against clear paper towels to remove any remaining liquid, and the plates were allowed to air dry for 3 hours at RT. Dried plates were wrapped in parafilm and stored at 4 °C for later use. To remove the stabilizing agent coating, wells were washed with slightly acidic distilled water (pH of 6) three times, leaving the plate prepared for subsequent assay steps.

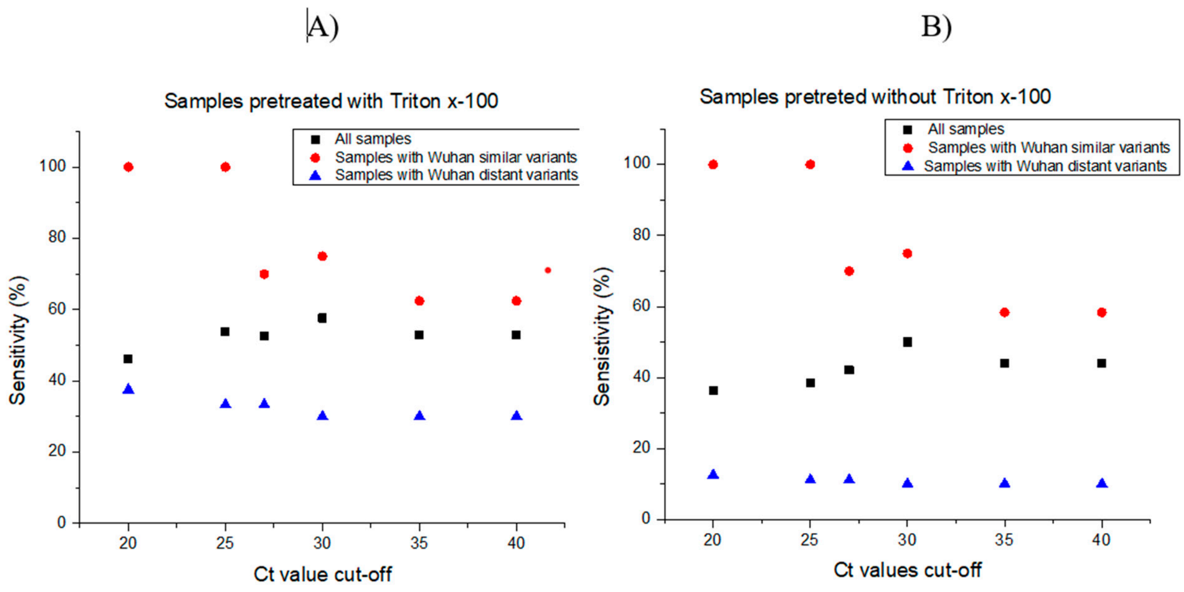

Figure S4. Dependence of N protein ELISA sensitivity on Ct value cut-off for samples treated with Triton X-100 (A) and samples without Triton X-100 pretreatment (B).

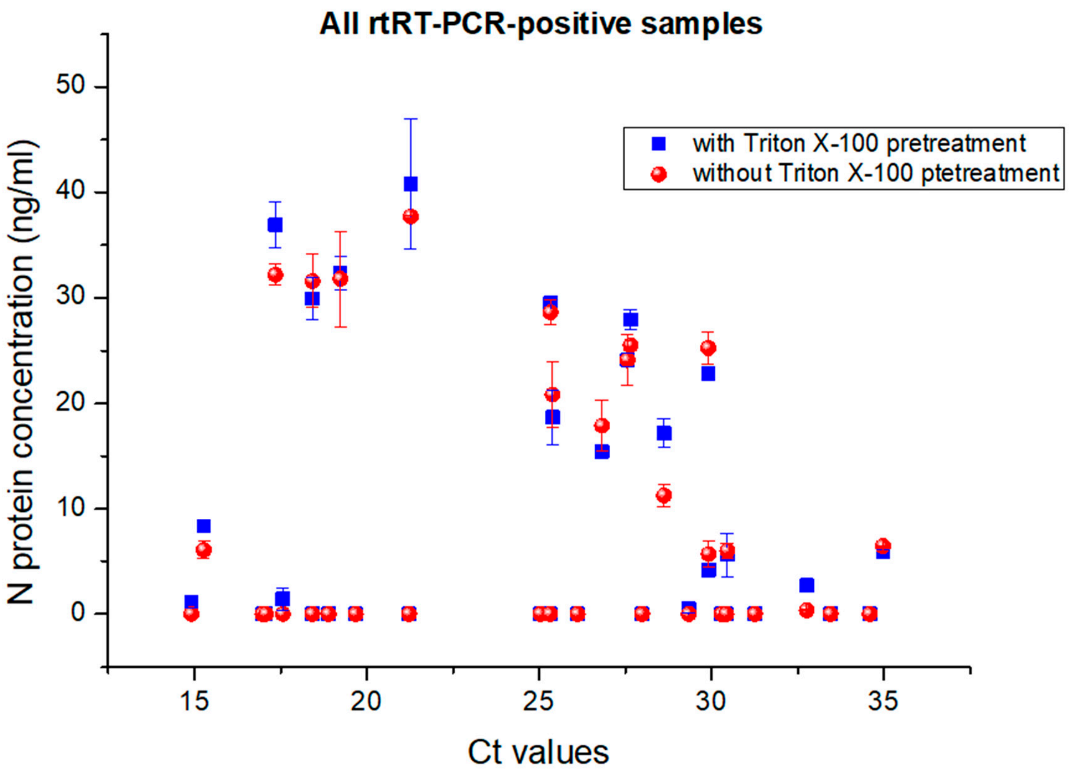

Figure S5. Distribution of N protein concentration in all rtRT-PCR-positive samples plotted against Ct values with and without Triton x-100 pretreatment.

II Materials and methods

Section S4. Characterization of N protein by HRMS

S4.1. SDS PAGE and in-gel digestion

Characterization of the produced recombinant N protein was done by HRMS after its in-gel digestion. A total of 10 µg of purified protein(s) were loaded in a 0.5 cm wide well and after SDS-PAGE gel was stained with Coomassie Brilliant Blue R-250 (CBB). Protein gel bands were washed, reduced with dithiothreitol, and alkylated with iodoacetamide, followed by in-gel trypsin digestion[1] (Shevchenko et al. 2006) with some minor modifications. The amount of trypsin was leveled to a trypsin/sample ratio of 1:30 (w/w). The final concentration of MS-grade trypsin (diluted in 25 mM ammonium bicarbonate buffer) was 1 ng/µL. Sample clean-up was performed using zip tips HyperSep C18 (Thermo Fisher Scientific Inc., Bremen, Germany).

S4.2.Nano Liquid Chromatography-electron spray ionization coupled to tandem mass spectrometry (nLC-ESI-MS/MS)

The resulting tryptic, and semi-tryptic peptides were analyzed by high-resolution, liquid chromatography (LC)-tandem mass spectrometry (MS/MS). Tryptic peptides from in-gel digestion were chromatographically separated using an UltiMate™ 3000 RSLC nano liquid chromatographic system (Thermo Scientific Inc., Bremen, Germany) and 2-column set up: a trap column C18, 50 mm (P/N 160454 Thermo Fisher Scientific) and analytical column PepMap C18, 15 cm × 75 μm, 3 μm particles, and 100 Å pore size (ES800A, Thermo Fisher Scientific, Waltham, MA, USA). The mobile phases were (A) water (MS-grade) with 0.1% formic acid and (B) acetonitrile (MS-grade) with 0.1% formic acid. The gradient program was as follows: 0–0.5 min 95% A, 0.5–10 min 95-66% A, 10–15 min 66-0% A, 15–20 min 0% A, 20–23 min 5% A, with a flow rate of 0.25 μL/min. Injection volume was 1 μL (150-200 ng of protein). This nLC system was coupled with an Orbitrap Exploris 240 mass spectrometer (Thermo Fisher Scientific Inc., Bremen, Germany) equipped with a heated electrospray ionization source. Analysis was performed in positive ion mode. Parameters of the ion source were as follows: spray voltage 1.9 kV, capillary temperature 300 °C, range 350–1600 m/z, resolving power 60 000, 1 micro scan was acquired using Xcalibur (version 4.4) software (Thermo Fisher Scientific) with the precursor mass tolerance of 10 ppm.

S4.3. Protein Database and PEAKS X Pro Studio Search Parameters for protein and modification identifications

The identification of proteins was performed by PEAKS Suite X (Bioinformatics Solutions Inc., Canada). Signature MS/MS spectra were searched using the PEAKS database (DB) and post-translational modification (PTM) algorithms against a database consisting of 7041 sequences from severe acute respiratory syndrome coronavirus 2 (2019-nCoV) (SARS-CoV-2) [2697049] UniProtKB, downloaded on 16/01/2023 from http://www.uniprot.org/, and the common repository of adventitious proteins (crap) contaminant database (downloaded on 01/03/2019 from https://www.thegpm.org/crap/).

S4.4. N protein post-translational modification (PTM) profiling

In the PEAKS DB algorithm, the following modifications were taken into account as variables: oxidation (Met), deamidation (Gln, Asn), and hydroxylation (Pro), while carbamidomethylation (Cys) was set as a fixed modification. In the PEAKS PTM algorithm modification, an open search was done on 313 inbuilt modifications updated from the UniMod database. Up to three missed trypsin cleavages were allowed per peptide. A semi-specific mode of trypsin cleavage was chosen, enabling tryptic and semi-tryptic peptide detection. Mass tolerances were set to 10 ppm for parent ions and 0.02 Da for fragment ions. Protein filters were set to protein -10 log P≥20, proteins' unique peptides ≥2, and an AScore of at least 50 for confident PTM identification. The peptide filter was set to the false discovery rate <0,1%. All spectra of modifications shown in Figure S1 and Table S1 were manually checked and if no experimental data supporting the finding, it was filtered out.

Section S5. Antisera production

S5.1 Immunization of rabbits and mice

Mice immunization

Swiss Webster mice (n=10) were immunized subcutaneously with N protein formulated with Complete Freund`s adjuvant (CFA; 1$^{st}$ dose, 100 µg N protein / dose) or Incomplete Freund`s adjuvant (IFA; 2nd and 3rd doses, 50 µg N protein / dose) in three-week intervals.

Mice were housed in small groups of up to six animals and had access to commercial mice food and water ad libitum.

N protein solution (500ug/ml in PBS) was sterilized by filtering through 0.22 um filters. Sterile N protein solution was mixed with CFA (Sigma, Cat. No. F5881) at ratio 1:1 (v/v) under aseptic conditions. In total 400 ul of N protein-CFA emulsion (N protein final concentration 250ug/ml) was applied per immunization per mouse.

Initial immunization was done by injection of N protein in CFA given subcutaneously (SC) in four sites (thigh pocket, base of tail, and mediastinum) with a 100 ul using 23-25 gauge needle. In total 100 ug of N protein was applied per mouse (25 ug per site).

Subsequent immunizations with booster doses were done in the same way, but using IFA (Sigma, Cat. No. F5506) instead of CFA and N protein final concentration was 125 ug/ml. . In total 50 ug of N protein was applied per mouse (12.5 ug per site). Immunizations were done every three weeks.

Mice immunization scheme:

1. day 0 – N protein in PBS: CFA = 1:1 (v/v); N protein final concentration was 250 µg/mL; 400 µL per mice (4x100 µL), e.g. 100 µg per mice

2. day 21 - N protein in PBS: IFA = 1:1 (v/v); N protein final concentration was 125 µg/mL; 400 µL per mice (4x100 µL), e.g. 50 µg per mice

3. day 42 - N protein in PBS: IFA = 1:1 (v/v); N protein final concentration was 125 µg/mL; 400 µl per mice (4x100 µL), e.g. 50 µg per mice

First bleeding was performed two weeks after the 3rd dose, and then in intervals not shorter than two weeks. The sera obtained after the first bleeding was tested for the production of specific anti-N protein antibodies.

Mice blood collection

Mouse blood samples for production of antiserum were collected by bleeding from retro-orbital sinuses. Prior bleeding mice were anesthetized (intraperitoneal injection of ketamine (˜90 mg/kg bw) /xylasine (˜10 mg/kg bw) in physiological solution). Blood samples were allow to clot for 30-60 min at RT. Clots were remouved by centrifugation at 2000 x g for 10 minutes at 4 °C. Upon centrifugation, supernatants (serum) were collected, pooled, decomplemented (1h, 56 °C), and stored at -20 °C.

Before the first immunization serum samples were taken as the reference point

Two weeks after three immunization (1 immunization plus two boosters, day 56) the blood (50 ul per mice) was collected to check the level of production of N-protein specific antibodies.

The first collection: At day 58 about 300 ul of blood was collected per mice (in total 3 ml of blood). Mice sera were pooled (1.5 ml).

The second collection: At day 72 (eg. 30 days from the final booster immunization) about 800 ul of blood was collected per mice (in total 3 ml of blood). Mice sera were pooled (4 ml).

Rabbit immunization

Two New Zealand White rabbits (n=2) were immunized with N protein formulated with CFA (1st dose) or IFA (2nd and 3rd doses) subcutaneously in four sites (1 mg N protein/dose) in two-week intervals. Rabbits were housed in small groups of up to six animals and had access to commercial rabbit food and water ad libitum.

Before the first immunization serum samples were taken as the reference point.

N protein solution (2 mg/ml in 2x PBS) was sterilized by filtering through 0.22 um filters. Sterile N protein solution was mixed with CFA (Sigma, Cat. No. F5881) at ratio 1:1 (v/v) under aseptic conditions. In total 1 ml of N protein-CFA emulsion was applied per immunization per rabbit.

Initial immunization was done by injection of antigen (N protein) in CFA given subcutaneously (SC) in four sites (2 sites at the neck and 2 sites at the flank) with a 0.25 ml per site using 23-25 gauge needle. In total 1.0 mg of N protein was applied per rabbit (250 ug per site).

Subsequent immunizations with booster doses were done in the same way, but using IFA (Sigma, Cat. No. F5506) instead of CFA. Immunizations were done every two weeks.

Animals are monitored immediately following injection for anaphylactic reactions, both after the primary injection and after the subsequent booster injections. Thereafter animals are monitored daily for inflammatory responses, granulomas or ulcerative lesions at the injection site(s) and for general behaviour.

Rabbit immunization scheme:

1. day 0 – N protein in PBS: CFA = 1:1 (v/v); N protein final concentration was 1 mg/mL; 1 mL per rabbit (4x250 µL), e.g. 1 mg per rabbit

2. day 14 – N protein in PBS: IFA = 1:1 (v/v); N protein final concentration was 1 mg/mL; 1 mL per rabbit (4x250 µL), e.g. 1 mg per rabbit

3. day 28 - N protein in PBS: IFA = 1:1 (v/v); N protein final concentration was 1 mg/mL; 1 mL per rabbit (4x250 µL), e.g. 1 mg per rabbit

Rabbit blood Collection

The first bleeding was performed two weeks after the 2nd dose, and the final blood collection was done three weeks after the 3rd dose. The sera obtained after the first bleeding was tested for the production of specific anti-N protein antibodies.

Blood collection was done from the right or left ear veins via a 22 gauge catheter and transferred into Falcon tube. Blood samples were allow to clotot for 30-60 min at RT. Clots were removued by centrifugation at 2000 x g for 10 minutes at 4 °C. Upon centrifugation, supernatants (serum) were collected, pooled, decomplemented (1h, 56 °C), and stored at -20 °C.

Pre-immunization blood sample (about 5 ml) was collected before the primary immunization, to ensure that antibodies to the antigens do not already exist.

The first collection: Two weeks after two immunization (1 immunization plus 1 booster, day 28), 12 ml of blood was collected from each of two rabbits (in total 24 ml), and 11 ml of sera was obtained.

The second collection: The final blood collection was done at day 62 (eg. 20 days from the final booster immunization) and about 30 ml of blood was collected from each of two rabbits (in total 60 ml), and 30 ml of sera was obtained.

Collected sera were centrifuged at 4000 g during 30 min, at 4 C. Top coat of remained lipids was carefully removed and sera were separated.

S5.2 Purification of antibodies from animal sera

Ammonium sulfate fractionation of antibodies

In this study, we employed a multistep protocol for the purification of serum samples. The detailed protocol is described in the following text. The obtained mice and rabbit sera were subjected to centrifugation at 4000 g for 30 minutes at 4 °C. The top layer containing residual lipids was meticulously removed. The volume of the resulting serum was measured. A saturated $(NH_4)_2SO_4$ (4.1 M at RT) solution was prepared, and the pH was adjusted to 7. The $(NH_4)_2SO_4$ solution was added in a dropwise manner to the serum with continuous stirring. The volume of $(NH_4)_2SO_4$ added equaled the serum volume. The solution was left overnight at 4°C with constant stirring for precipitation. An aliquot was withdrawn for subsequent analyses. The next day, the solution was centrifuged at 3000 g for 30 minutes at 4°C. The supernatant was separated from the pellet, and an aliquot of the supernatant was collected for further analysis. 1.25 mL of phosphate-buffered saline (PBS) was added per mL to the puffy pellet to solubilize it, resulting in approximately 1.625 mLl of solubilized pellet. An aliquot of the solubilized pellet was withdrawn for further analysis. The solubilized pellet underwent extensive dialysis against 4 L PBS using a dialysis tube with cut-off 10 kDa at 4°C for 72 hours, involving three buffer exchanges. The dialyzed solution was collected, and the dialysis tube was washed with a small volume of PBS. The volume of the collected solution was measured. The dialyzed solution was mixed with an equal volume of tetrachloroethylene and emulsified. The emulsion was then centrifuged at 4500 g for 10 minutes at 4°C. The top clear supernatant was separated from the middle layer of precipitated proteins and the bottom tetrachloroethylene layer. The volume of the clear supernatant was measured. The volume of the clear supernatant was adjusted to match the initial serum volume after lipid removal. An aliquot of this adjusted supernatant was withdrawn for further analysis. Protein concentration

was assessed using UV-VIS spectroscopy by measuring absorbance at 280 nm and by the BCA assay.

Antibody purification on Protein A Sepharose

The following protocol was employed for the purification of rabbit IgG antibodies from serum. Protein A Sepharose 98% (HPLC and SDS-PAGE) from Roche, Basel, Switzerland was used as a purification medium. Starting buffer (PBS, pH 7.4), elution buffer (0.1 M glycine acid, pH 2.8), and Tris buffer for neutralization (1.5 M Tris pH 8) were prepared. At first, the matrix was rinsed in a tube with water to remove the 20% ethanol storage solution, followed by centrifugation at 800 g for 5 minutes. After centrifugation, 3 mL of matrix was packed into a 5 mL syringe. The packed matrix was equilibrated with min 20 volumes of the starting buffer (PBS). Rabbit sera (5 mL, 125 mg of total protein) was diluted with 1 volume of PBS (10 mL in total). The diluted sera were applied to the column at a low flow rate. The flowthrough was reapplied to the column, and this step was repeated three times (3x sample load). The last flowthrough was saved. Unbound serum proteins were washed until a negative Bradford reaction using approximately 50 mL of the loading buffer (about 17 column volumes). In tubes for collecting bound proteins, 200 µL of Tris buffer was added for immediate neutralization upon collection (20% of the total volume of the eluted fraction, which was 1 mL). The bound proteins were eluted with an elution buffer until a negative Bradford reaction (16 tubes in total were collected). The purity of the collected fractions was analyzed by SDS-PAGE. Fractions with pure IgGs were pooled and dialyzed against 4 L of PBS (cut off 10 kDa) for 72 hours at 4°C, with constant stirring and three buffer exchanges. After dialysis, IgG protein concentration was determined, followed by EC50 determination in a direct ELISA.

Section 6. Compositions of simulated body fluids

Table S2 Composition of simulated serum[2]

Component	Concentration
**Human Serum Albumin (HSA) in phosphate buffered saline (PBS)**	**6% (w/v)**

Table S3 Composition of simulated salivary fluid[3]

Preparation of stock solutions of simulated salivary fluid. The volumes are calculated for a final volume of 500 mL for each simulated fluid. The recommendation is to make up the stock solution with distilled water to 400 mL instead, i.e. 1.25 concentrate, for storage at -20 C.

Constituent	Stock conc.		Vol. of stock	Conc. in SSF
	**g L $^{-1}$**	**mol L $^{-1}$**	**mL**	**mmol L -1**
**KCl**	**37.3**	**0.5**	**15.1**	**15.1**
**KH_2PO_4**	**68**	**0.5**	**3.7**	**3.7**
**$NaHCO_3$**	**84**	**1**	**6.8**	**13.6**

**NaCl**	**117**	**2**	**-**	**-**
**$MgCl_2(H2O)_6$**	**30.5**	**0.15**	**0.5**	**0.15**
**$(NH_4)_2CO_3$**	**48**	**0.5**	**0.06**	**0.06**

Table S4 Composition of simulated urine[4]

Constituent	Molarity (mM)	Quantity (g/100ml)
**Na_2SO_4**	**11.965**	**0.1700**
**$C_5H_4N_4O_3$**	**1.487**	**0.0250**
**$Na_3C_6H_5O_7x2H_2O$**	**2.450**	**0.0720**
**$C_4H_7N_3O$**	**7.791**	**0.0881**
**CH_4N_2O**	**249.750**	**1.5000**
**KCl**	**30.953**	**0.2308**
**NaCl**	**30.053**	**0.1756**
**$CaCl_2$**	**1.663**	**0.0185**
**NH_4Cl**	**23.667**	**0.1266**
**$K_2C_2O_4xH_2O$**	**0.19**	**0.0035**
**$MgSO_4x7H_2O$**	**4.389**	**0.1082**
**$NaH_2PO_4x2H_2O$**	**18.667**	**0.2912**
**$Na_2HPO_4x2H_2O$**	**4.667**	**0.0831**

7. References

(1) Shevchenko, A.; Tomas, H.; Havliš, J.; Olsen, J. V.; Mann, M. In-Gel Digestion for Mass Spectrometric Characterization of Proteins and Proteomes. *Nat Protoc* **2007**, *1* (6), 2856–2860. https://doi.org/10.1038/nprot.2006.468.

(2) van de Merbel, N. C. Quantitative Determination of Endogenous Compounds in Biological Samples Using Chromatographic Techniques. *TrAC Trends in Analytical Chemistry* **2008**, *27* (10), 924–933. https://doi.org/https://doi.org/10.1016/j.trac.2008.09.002.

(3) Minekus, M.; Alminger, M.; Alvito, P.; Ballance, S.; Bohn, T.; Bourlieu, C.; Carrière, F.; Boutrou, R.; Corredig, M.; Dupont, D.; Dufour, C.; Egger, L.; Golding, M.; Karakaya, S.; Kirkhus, B.; Le Feunteun, S.; Lesmes, U.; MacIerzanka, A.; MacKie, A.; Marze, S.; McClements, D. J.; Ménard, O.; Recio, I.; Santos, C. N.; Singh, R. P.; Vegarud, G. E.; Wickham, M. S. J.; Weitschies, W.; Brodkorb, A. A Standardised Static in Vitro Digestion Method Suitable for Food-an International Consensus. *Food Funct* **2014**, *5* (6), 1113–1124. https://doi.org/10.1039/c3fo60702j.

(4) Sarigul, N.; Korkmaz, F.; Kurultak, İ. A New Artificial Urine Protocol to Better Imitate Human Urine. *Sci Rep* **2019**, *9* (1). https://doi.org/10.1038/s41598-019-56693-4.
